# Enoxaparin versus Unfractionated Heparin for the Perioperative Anticoagulant Therapy in Patients with Mechanical Prosthetic Heart Valve Undergoing Non-Cardiac Surgery

**DOI:** 10.3390/medicina58081119

**Published:** 2022-08-18

**Authors:** Luminita Iliuta, Andreea Andronesi, Georgiana Camburu, Marius Rac-Albu

**Affiliations:** 1 Department of Medical Informatics and Biostatistics, University of Medicine and Pharmacy “Carol Davila”, 050474 Bucharest, Romania; 2 Cardioclass Clinic for Cardiovascular Disease, 031125 Bucharest, Romania; 3 Nephrology Department, University of Medicine and Pharmacy “Carol Davila”, 050474 Bucharest, Romania; 4 Nephrology Department, Fundeni Clinical Institute, 022328 Bucharest, Romania

**Keywords:** mechanical prosthetic valves, anticoagulation, low-molecular-weight heparin, unfractionated heparin, enoxaparin, non-cardiac surgery

## Abstract

*Background and Objectives*: Immediate postoperative anticoagulation regimens in patients with mechanical prosthetic valves undergoing non-cardiac surgery are clear only for unfractionated heparin (UH), whereas the few low-molecular-weight heparin (LMWH) trials available to date concern the use of Enoxaparin in general/orthopedic surgery. We performed a single-center real-world data study comparing the efficacy and safety of LMWH—Enoxaparin (E)— and UH during the perioperative period in non-cardiac surgical procedures in patients with mechanical prosthetic valve replacement in the mitral, aortic, or tricuspid positions. *Materials and Methods*: We enrolled 380 patients, who received E or UH together with oral anticoagulation with antivitamin K (acenocoumarol) until they achieved an optimal International Normalized Ratio (INR). Objective assessment of E efficacy included the following: normal value for all the parameters of ultrasound prosthetic functioning, no early thrombosis of the prosthesis, and rapid achievement of target INR with a decreased period of subcutaneous anticoagulation. Subjective assessment included the following: clinical improvement with decreased immobilization and in-hospital stay, fewer gluteal ulcerations, and fewer postoperative depression and anxiety episodes. *Results*: Comparing with UH, anticoagulation with E was more effective (*p* < 0.0001 and *p* = 0.02). The probability of death was smaller in the E group compared with the UH group. No major hemorrhagic event was reported. Mild bleeding episodes and thrombocytopenia were more common in the UH group. Patient’s compliance and quality of life were better with E due to shortened hospitalization, decreased need for testing of coagulation (every 6 h for UH), better dosing (SC every 12 h for E versus continuous infusion for UH), shortened immobilization during the immediate postoperative period with subsequent improvement in the psychological status, as well as due to lack of significant side effects. *Conclusions*: Taking into consideration the improved efficiency and safety, as well as all the supplementary advantages, such as no need for anticoagulation monitoring, the ease of administration, and reduced duration of hospitalization, E should be seen as an attractive alternative for anticoagulation which deserves further investigation.

## 1. Introduction

The management of anticoagulation in patients with a mechanical prosthetic heart valve who are receiving long-term oral anticoagulant therapy and are undergoing elective non-cardiac surgery can be especially difficult and problematic [1,2]. Temporary discontinuation of anticoagulants increases the risk of valve thrombosis and systemic embolism [1,2,3,4]. On the other hand, continuing anticoagulants perioperative, or stopping and restarting anticoagulants too soon after surgery, can cause life-threatening bleeding [5]. The optimal anticoagulation strategy has to minimize the risk of thromboembolism, without causing excessive postoperative bleeding [6,7,8].

The pharmacokinetic and pharmacodynamic properties of low-molecular-weight heparins (LMWH) [9,10,11,12] are attractive compared to other anticoagulant strategies, but unfortunately there are not enough studies to take into account their safety and effectiveness for the anticoagulation of mechanical heart valve prosthesis. Unlike unfractionated heparin (UH), LMWH have further predictable kinetics, are less protein bound, have lower potential for platelet activation and bear no monitoring, so they are a better alternative for the perioperative anticoagulation in patients with mechanical heart valves undergoing non-cardiac interventions [9]. The few studies of LMWH used in general surgery, specifically in patients with heart valve prosthesis, have shown a decrease in the incidence of prosthesis obstructive dysfunctions, as well as an increase in postoperative convenience and the quality of life in LMWH versus UH patients [12,13,14,15,16]. Large-scale studies of LMWH given in the perioperative anticoagulant therapy of mechanical prosthetic valve patients undergoing general surgery interventions have never been conducted before.

## 2. Materials and Methods

We performed a real-world data study with open study period, which included 380 consecutive patients from a single center with mechanical prosthetic heart valves in mitral, aortic or tricuspid positions/combinations of the three, who underwent noncardiac surgery interventions between 1 January 2017 and 1 January 2020. Non-eligibility criteria were listed in Table 1. Study drop-out criteria were severe thrombocytopenia, active bleeding. The essential inclusion criteria (gender, age, diagnosis), the duration of treatment and assessment criteria were similar in the two treatment groups (*p* < 0.0001). The protocol was approved by the institute management, and every patient signed the informed consent form.

**Figure 1:**
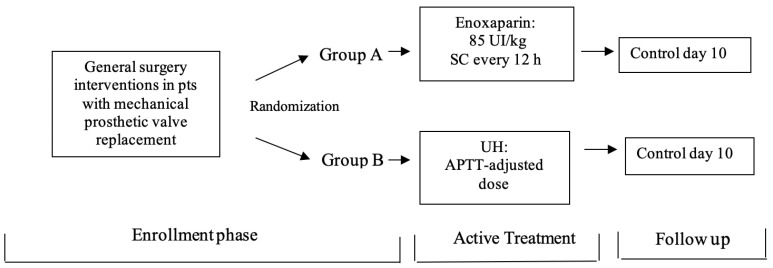
Study protocol phases. SC—subcutaneous; UH—unfractionated heparin; APTT—activated partial thromboplastin time.

Patients were randomized (using stratified randomization method) to receive enoxaparin or UH as antithrombotic treatment (Figure 1): Group A—Enoxaparin (E) SC 85 IU/kg twice daily (every 12 h); Group B—Unfractionated heparin (UH) given as IV continuous infusion of doses adjusted to maintain the APTT at 2.5 the normal value. The treatment was given for a period of no less than 72 h and up to 10 days. We measured INR after admission the first hours and before starting the enoxaparin treatment. INR was considered correct if it was in the target range of the recently published recommendations in managing oral anticoagulation for the valvular prosthesis type, or incorrect if it was not [17]. For each patient, we prospectively determined the following: demographic details, the reason for stopping acenocumarol, the global risk for thromboembolic events, INR before starting enoxaparin/UH, days number with enoxaparin/UH and mean anti-Xa activity level during treatment and the appearance of hemorrhagic or thromboembolic events during hospitalization and follow-up.

The bleeding events were classified as minor events (which did not require additional testing requirement), and major events (life-threatening or fatal bleeding episodes needing transfusion or hospitalization).

We assessed clinical and laboratory parameters both at baseline and at the end of the treatment. From clinical point of view, we measured: NYHA class for heart failure, clinical parameters for the prostheses, patient compliance and quality of life. Laboratory parameters included: the usual blood tests, echocardiographic measurements of the prosthetic valves and clotting tests (INR for both arms and APTT for arm B) [18]. Based both on clinical criteria and through thoracic and transesophageal echocardiography we diagnosed early development of prosthetic thrombosis.

Enoxaparin dosing was performed according to patients’ kidney function. In those with normal estimated glomerular filtration ratio (eGFR), the standard enoxaparin dose was 1 mg/kg administered subcutaneously twice a day once the INR was below 1.5. Patients with serum creatinine ≥ 1.5 mg/dL (≥133 μmol/L) received the lowest dose of E required to obtain an anti-Xa activity level above 0.4 IU/mL [18]. Enoxaparin was withheld in all patients 12–18 h before the procedure and restarted with the same schedule after the procedure once hemostasis was achieved.

In patients with a bleeding episode, we stopped acenocumarol and started enoxaparin at the prophylactic dose of 40 mg/day, immediately after INR was below 1.5. Additionally, we kept Enoxaparin until hemostasis was achieved, and we considered that the risk of new bleeding episodes is low, after which we administrated the standard dose of 1 mg/kg two times daily. We restarted Acenocumarol at the patient’s usual dosage a few days before discharge, and we stopped enoxaparin when INR became > 2.

We measured anti-Xa activity six hours after administration of enoxaparin and, after that, depending on the level of anti-Xa activity, new measurements were taken at 3-day intervals. Patients were followed up using telemedicine application/telephone, clinical evaluation and echocardiography during hospitalization, and in the first three months after surgery.

The primary endpoints were the composite of 30-day mortality, in-hospital prosthesis obstructive dysfunction–thrombotic obstruction (safety endpoints), duration of hospital stay and immobilization, and the above endpoint plus in-hospital intracranial hemorrhage or in-hospital major bleeding other than intracranial bleeding (primary-efficacy-plus-safety endpoint). Safety data were reported monthly to the safety monitoring committee. Safety data were reported monthly, and stroke cases were evaluated by an independent commission in which the members were blinded of treatment assignment. The data were collected and processed using the Visual Fox Pro Excel, EpiInfo, Systat, and SPSS programs. We defined a detailed analysis plan before the database was locked, but with no confirmatory pre-specified statistical hypothesis. For categorical data we used numbers and percentages, and for continuous data mean ± standard deviation or median (interquartile range) as appropriate. For the pairwise comparisons of primary interest, we generate risk ratios and CIs (CI = confidence index) which were presented with the two-sided 95% CI of the relative risk and with normal *p* values. For the primary endpoints the study groups were compared using Kaplan–Meier curves and log-rank tests. Additionally, a two-sided 95% CI was calculated for each endpoint, and the treatment groups were compared using an overall chi-square test, alpha level value of 0.05. The frequency of the primary-efficacy-plus-safety endpoint for the UH group as a reference group was 17.7%. Taking into account the basis of phase-II studies, we assumed that the experimental group with E would result in better, or at least similar outcomes when compared with standard treatment. Therefore, based on the non-inferiority of the experimental group versus the reference group, we performed the sample size and power calculations. The study’s power to exclude was 80%, with 95% one-sided confidence. Compared with the reference group, we found in the experimental treatment group 1% higher rate for the primary endpoints, 1.7% lower rate for the efficacy endpoint and 2% lower rate for both the efficacy and safety endpoint.

## 3. Results

We found similar baseline characteristics in the two groups (Table 2).

**Figure 2:**
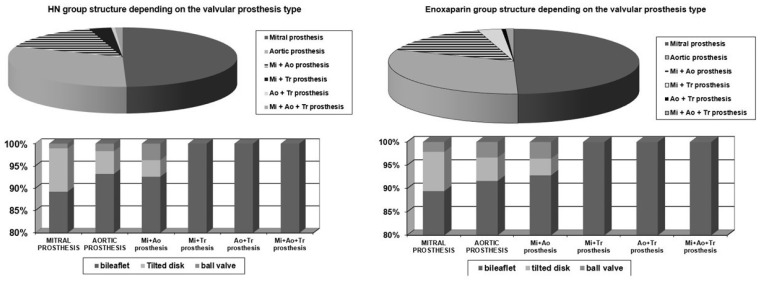
Valvular prosthesis type in the study groups.

Depending on the type of the valve prosthesis, the two groups were homogenous (Figure 2).

There were no significant differences between the study groups concerning in-hospital concomitant medications (Table 3). The proportion of patients receiving antiplatelet therapy with Aspirin, Ticlopidine, or Clopidogrel was 7.81% in group A and 7.45% in group B.

The primary efficacy and efficacy-plus-safety endpoints and their components in the treatment groups are shown in Table 4.

**Figure 3:**
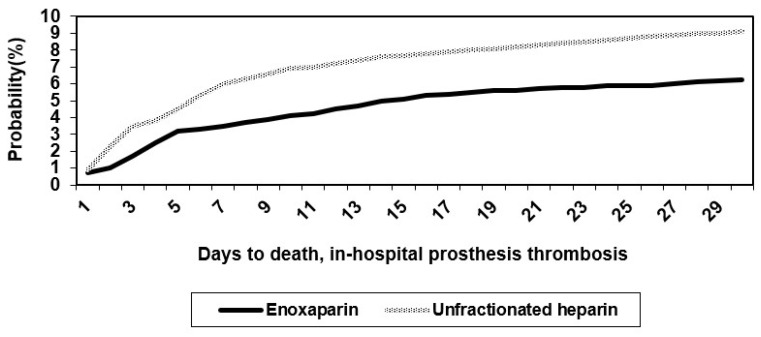
Kaplan–Meier curves for primary endpoints in the study groups.

The combined efficacy and safety outcome in the UH group of 20/188 patients (10.64%) was similar to that estimated before the trial commenced (11%). The Kaplan–Meier curves for these primary endpoints are shown in Figure 3.

Log-rank tests were highly significant. Early after treatment, the curve for the E started to separate from that of UH, and also, at 48 h, differences between the two groups in the primary endpoints were already present. For the primary efficacy endpoint, the event rates were 6.25% for the E group and 8.51% for the UH group (*p* < 0.0001). For the primary-efficacy-plus-safety endpoint, the rates were 6.25% for the E group and 9.04% for the UH group (*p* = 0.02). The relative risks in the two groups are presented in Table 5.

**Figure 4:**
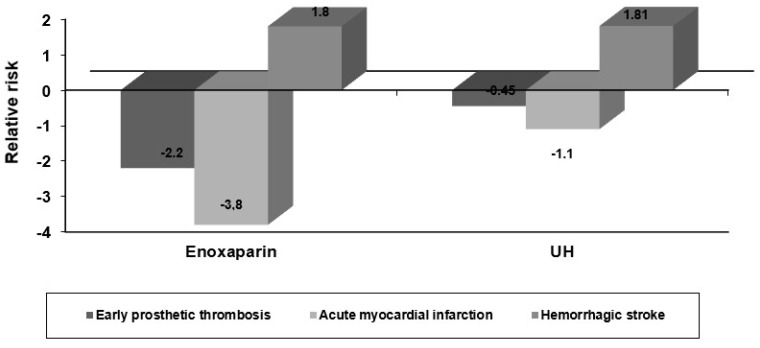
Early major complications in the study groups.

The composite endpoints rates were lower in the group of patients treated with E compared with patients treated with UH. The *p* values resulting from conventional statistical testing for E versus HN were statistically significant, being 0.0002 and 0.0003, respectively, for the primary-efficacy-plus-safety composite endpoints. Additionally, in patients treated with E, in-hospital prosthesis thrombosis occurred less frequently than in those treated with UH. The in-hospital death rates were lower in the E group (6.25%) compared to UH group (8.51%). No significant differences in other major cardiovascular complications were seen, with the exception of a significantly lower rate for postoperative myocardial infarction in patients with risk factors in the E versus the UH group (Figure 4).

The mean duration of hospitalization in the E group was 10.5 ± 4.3 days, compared to 14.5 ± 5.8 days in the UH group. The mean immobilization interval during the immediate postoperative period in group A versus group B was 1.02 ± 3 days versus 5.5 ± 2.8 days, respectively.

Concerning the complications of anticoagulant therapy, the in-hospital strokes data are summarized in Table 6.

We found similar rates for total stroke and intracranial hemorrhage in the two study groups with a few number of hemorrhagic conversions in each of these. The rates of non-cerebral bleeding complications, number of patients needing transfusions and the thrombocytopenia rates are presented in Table 7.

**Figure 5:**
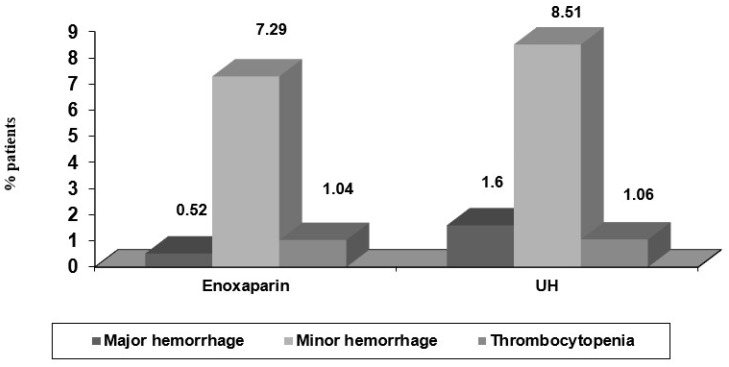
Incidence of bleeding complications and thrombocytopenia in the study groups.

In patients with antiplatelet associated treatment with Aspirin, Ticlopidine or Clopidogrel, we found significantly more major bleeding complications (*p* = 0.0001), more need for transfusions (*p* = 0.002) and a higher rate of thrombocytopenia (*p* = 0.001). Additionally, in patients older than 75 years and in diabetics, the rate of major bleeding complications was three times higher in those with associated antiplatelet therapy (4% versus 14% and 2% versus 7%, respectively). Although the differences were not significant, we found more major and minor bleeding complications in the UH group compared with E group, with no more episodes of thrombocytopenia in the E group (Figure 5).

The total number of re-hospitalizations was similar in the two treatment groups (11.41% in the E group, 11.7% in the UH group) with a few additional strokes occurring after hospital discharge in the two groups. The probability of death, early prosthetic thrombosis and perioperative myocardial infarction were smaller with E versus UH. No significant changes in liver function tests and blood biochemistry were recorded in the groups.

## 4. Discussion

Despite the high global risk of thromboembolism in our study, during hospitalization or follow-up (with a mean of 2.8 months), no thromboembolic event was detected. Although the method used during the follow-up for detecting all the thromboembolic events (particularly valve thrombosis) may have limitations, we think it is sensitive enough for the detection of those with clinical or ecographic signs, who are reported mostly in other studies.

Regarding safety, we observed only one important bleeding complication in the E group, but we have to take into account that most of the procedures were in low-to-medium bleeding risk, and the mean levels of anti-Xa activity were in the lower limit of the therapeutic range (95% of patients had anti-Xa activity < 1.1 IU/mL). In patients who bled during E treatment, the highest anti-Xa activity was 1.1 IU/mL.

E has shown increased efficacy over UH in the perioperative anticoagulation in general surgery interventions of mechanical prosthetic heart valve patients. Subjective measures in patients achieving a more favorable response included the improvement of clinical status with decreased hospitalization time, decreased immobilization and so fewer gluteal ulcerations and fewer postoperative episodes of depression and anxiety. Objective measures of E efficacy in patients with mechanical prosthetic heart valves included the following: maintenance of all ultrasound prosthetic functioning parameters within the normal range, absence of early prosthesis thrombosis in the study group, decreased parenteral anticoagulation period with rapid achievement of optimal INR. The probability of death was smaller in the E group compared with the UH group. No major hemorrhagic event was reported in either of the two groups. In the UH group, we found more episodes of minor hemorrhage and thrombocytopenia. Additionally, patients’ compliance and quality of life were improved due to shortened hospital stay, fewer coagulation tests (every 6 h for UH), increased dosing convenience (SC every 12 h for E versus continuous infusion for UH), shortened immobilization during the immediate postoperative period with subsequent improvement in the psychological status, as well as the lack of significant side effects.

### Limitations

First, although this is one of the largest studies using E for perioperative anticoagulation in patients with mechanical valve prosthesis, the number of patients was still moderate. Second, data on thromboembolic events (particularly valve thrombosis) were obtained from clinical evaluation and transthoracic echocardiography rather than transesophageal eco. However, the cardiologists performing these examinations were experienced in evaluating patients with valve prostheses. Third, the study design and the number of patients did not allow conclusions about the efficacy and safety of E in these patients compared with other methods. Taking into account that our patients were not selected, our results represent a real clinical practice observation of using E perioperatively in mechanical heart valves patients.

## 5. Conclusions

Considering efficacy and safety and additional advantages, such as the ease of administration, lack of need for long-term monitoring of anticoagulation, and reduction in the hospitalization duration, E should be regarded as an attractive alternative pharmacological anticoagulation strategy in patients with mechanical heart valves undergoing noncardiac interventions, thus deserving further study.

## Figures and Tables

**Table 1:** Non-eligibility criteria.

Non-Eligibility Criteria
Actual peptic ulcer of the stomach or duodenum
Known hypersensitivity to fractionated and unfractionated heparin
Conditions associated with increased bleeding risk (coagulation disorders, coagulation factor deficiencies, thrombocytopenia, serious liver or renal disorders).
Cerebral/other trauma within the previous 24 h.
General surgery or organ biopsy within the last 2 months.
History of stroke, transient ischemic attack or dementia, or any known organic CNS disease.
Thrombocytopenia < 100,000 cells/μL
Chronic kidney disease (serum creatinine > 221 μmol/L in men and > 177 μmol/L in women)
Cardiopulmonary resuscitation for more than 10 min within the past 2 weeks
Pregnancy or breastfeeding or birth within the last 30 days
Participation in another study
Absence to follow-up visits or failure to comply with the study protocol
Actual peptic ulcer of the stomach or duodenum
Known hypersensitivity to fractionated and unfractionated heparin

CNS—central nervous system.

**Table 2:** Baseline characteristics of the study groups.

	Group A—192 pts	Group B—188 pts
Mean (SD) age (years)	51 (12)	51 (13)
Age > 70 years	13.02%	12.76%
Women	62.5%	63.8%
Mean (SD) weight (kg)	66 (15)	66 (14)
Mean (SD) height (cm)	170 (9)	170 (10)
NYHA class I/II	15.62%	15.96%
NYHA class III	72.91%	73.94%
NYHA class IV	11.46%	10.11%
Mean (SD) systolic blood pressure (mmHg)	134 (22)	133 (23)
Mean (SD) heart rate/atrial fibrillation	75 (17)/52.08%	74 (17)/52.66%
Hypertension	10.42%	10.11%
Diabetes mellitus	5.73%	5.32%
Current smoker	4.69%	5.32%
Re-intervention (previous valve prosthesis)	9.89%	10.64%

NYHA—New York Heart Association.

**Table 3:** Concomitant cardiovascular medications during hospital stay in study groups (number of patients/percent).

	Enoxaparin Group	UH Group
Digoxin	101 (52.6%)	100 (53.19%)
ACE inhibitors	161 (83.85%)	159 (79.79%)
Angiotensin II inhibitors	2 (1.04%)	2 (1.06%)
Aspirin	10(5.21%)	9 (4.79%)
Ticlopidine/Clopidogrel	5 (2.6%)	5 (2.39%)
Beta blockers	88 (45.83%)	86 (45.74%)
Diuretics	126 (65.63%)	124 (65.96%)
Aldactone	84 (43.75%)	83 (44.15%)

ACE—angiotensin converting enzyme; UH—unfractionated heparin.

**Table 4:** Frequency of composite and single endpoints at hospital discharge and 30 days after discharge.

	Enoxaparin Group	UH Group
30-day mortality, in-hospital prosthesis thrombosis	14/192 (6.25%)	17/188 (9.04%)
30-day mortality, in-hospital prosthesis thrombosis or in-hospital major bleeding (other than intracranial hemorrhage)	13/192 (6.77%)	20/188 (10.64%)
Death at 30 days	12/192 (6.25%)	16/188 (8.51%)
In-hospital prosthesis thrombosis	0/192 (0%)	1/188 (0.53%)
In-hospital intracranial hemorrhage	1/192 (0.52%)	1/188 (0.53%)
Major bleeding (other than intracranial hemorrhage)	1/192 (0.52%)	3/188 (1.6%)
Hospitalization duration > 15 days	14/192 (0.52%)	100/188 (53.19%)
Immobilization for > 3 days	15/192 (7.81%)	123/188 (65.43%)
Gluteal ulceration	0/192 (0%)	4/188 (2.13%)

UH—unfractionated heparin.

**Table 5:** Relative risks and 95% CIs for primary efficacy composite endpoint in the two groups.

Death at 30 Days, in-Hospital Prosthesis Thrombosis, Hospitalization Duration > 15 Days, or Ulceration	Enoxaparin Group	UH Group	Relative Risk Enoxaparin Better	Relative Risk UH Better
Overall event rate	14/192 (7.29%)	17/188 (9.04%)	0.64	0.94
Age < 70 years	5/167 (2.99%)	7/164 (4.27%)	0.40	0.55
Age > 70 years	9/25 (36%)	10/24 (41.67%)	1.34	3.8
NYHA IV class	10/22 (45.46%)	11/19 (57.89%)	1.23	4.2
Type of prosthesis				
-bileaflet	8/175 (4.57%)	9/172 (5.23%)	0.33	0.34
-tilted disk	5/12 (41.67%)	6/13 (46.15%)	0.91	1.34
-ball valve	1/5 (20%)	2/3 (66.67%)	1.78	3.3
Prosthesis position				
-mitral	2/95 (3.11%)	3/93 (3.22%)	0.2	0.33
-aortic	2/60 (3.33%)	2/59 (3.39%)	0.3	0.3
-combined	10/37 (27.03%)	12/36 (33.34%)	0.9	1.2
Ulceration	0/192	4/188 (2.13%)		3.8

UH—unfractionated heparin; NYHA—New York Heart Association.

**Table 6:** In-hospital stroke rates.

	Enoxaparin Group	UH Group	*p* Value
Total strokes	3/192 (1.56%)	3/188 (1.59%)	0.95
Intracranial hemorrhage	1/192 (0.52%)	1/188 (0.53%)	0.92
Ischemic stroke (including hemorrhagic transformation)	2/192 (1.04%)	2/188 (1.06%)	0.78

UH—unfractionated heparin.

**Table 7:** Rates of in-hospital non-cerebral bleeding complications and thrombocytopenia.

	Enoxaparin Group	UH Group	*p* Value
Any thrombocytopenia	2/192 (1.04%)	2/188 (1.06%)	0.78
Thrombocytopenia			
<20,000 cells/μL	0	0	NA
20,000–50,000 cells/μL	1/192 (0.52%)	1/188 (0.53%)	0.92
50,000–100,000 cells/μL	1/192 (0.52%)	1/188 (0.53%)	0.92
Bleeding episodes			
Total	15/192 (7.89%)	19/188 (10.11%)	0.68
Major	1/192 (0.52%)	3/188 (1.6%)	0.63
Minor	14/192 (7.29%)	16/188 (8.51%)	0.71
Blood transfusion	7/192 (3.64%)	6/188 (3.19%)	0.85

NA—not applicable; UH—unfractionated heparin.

## Data Availability

All data generated or analyzed during this study are included in this published article.
